# Transmission-blocking activity induced by malaria vaccine candidates Pfs25/Pvs25 is a direct and predictable function of antibody titer

**DOI:** 10.1186/1475-2875-6-107

**Published:** 2007-08-08

**Authors:** Kazutoyo Miura, David B Keister, Olga V Muratova, Jetsumon Sattabongkot, Carole A Long, Allan Saul

**Affiliations:** 1 Malaria Vaccine Development Branch, National Institute of Allergy and Infectious Diseases, National Institutes of Health, USA; 2 Department of Entomology, United States Army Medical Component, Armed Forces Research Institute of Medical Sciences, Bangkok, Thailand

## Abstract

**Background:**

Mosquito stage malaria vaccines are designed to induce an immune response in the human host that will block the parasite's growth in the mosquito and consequently block transmission of the parasite. A mosquito membrane-feeding assay (MFA) is used to test transmission-blocking activity (TBA), but in this technique cannot accommodate many samples. A clear understanding of the relationship between antibody levels and TBA may allow ELISA determinations to be used to predict TBA and assist in planning vaccine development.

**Methods:**

Rabbit anti-Pfs25 sera and monkey anti-Pvs25 sera were generated and the antibody titers were determined by a standardized ELISA. The biological activity of the same sera was tested by MFA using *Plasmodium *gametocytes (cultured *Plasmodium falciparum *or *Plasmodium vivax *from malaria patients) and *Anopheles *mosquitoes.

**Results:**

Anti-Pfs25 and anti-Pvs25 sera showed that ELISA antibody units correlate with the percent reduction in the oocyst density per mosquito (Spearman Rank correlations: 0.934 and 0.616, respectively), and fit a hyperbolic curve when percent reduction in oocyst density is plotted against antibody units of the tested sample. Antibody levels also correlated with the number of mosquitoes that failed to become infected, and this proportion can be calculated from the reduction in oocyst numbers and the distribution of oocysts per infected mosquito in control group.

**Conclusion:**

ELISA data may be used as a surrogate for the MFA to evaluate transmission-blocking vaccine efficacy. This will facilitate the evaluation of transmission-blocking vaccines and implementation of this malaria control strategy.

## Background

The World Health Organization estimates that malaria causes 300–500 million clinical cases and 1 million deaths each year worldwide. Parasite strains that are resistant to anti-malarial drugs and mosquito vectors resistant to insecticides have emerged, enhancing the need for effective vaccines [1,2]. While multiple stages of the parasite life cycle are being targeted for vaccine development, vaccines against mosquito stage antigens are among the most novel. These mosquito-stage transmission-blocking (MSTB) vaccines are designed to prevent successful parasite infection of the mosquito vector and consequently prevent further parasite spread among humans [3].

In areas of low malaria transmission, MSTB vaccines, as a component of an integrated programme, may locally eliminate malaria transmission. Even in areas of high transmission the entomological inoculation rates (EIR) correlates with mortality rates, especially in infants [4], and by reducing transmission rates a MSTB vaccine may reduce the incidence of disease. A different form of transmission-blocking vaccine, the RTS, S vaccine that blocks transmission of sporozoites from mosquitoes to humans, has recently demonstrated significant reduction in both uncomplicated and severe malaria in trials in Mozambique [5] and similar effects on both transmission and disease incidence have been widely observed with use of bed nets [6-8]. In addition, transmission-blocking vaccines may prevent the spread of drug-resistant parasites or parasite mutants that have developed resistance to other malaria vaccines.

*Plasmodium falciparum *mosquito stage antigen Pfs25 and its homologue in *Plasmodium vivax*, Pvs25, are members of the P25 family of cysteine-rich 25 kDa antigens. They are composed of four tandem epidermal growth factor-like domains and are expressed on zygotes and mature ookinete stages of parasites within mosquitoes [9-11]. Because P25 is only expressed in the mosquito midgut and not in the vertebrate host, these proteins have not been under selection pressure by the host immune system and antigenic variation of P25 appears to be more limited than most vaccine candidates present in pre-erythrocytic and asexual blood stages [11-13].

An ex vivo assay, the mosquito membrane feeding assay (MFA), has been used to evaluate vaccines directed against mosquito stage antigens by measuring the transmission-blocking activity of the resulting antibody. In this assay, a mixture of a test serum and malaria gametocytes are fed to susceptible mosquitoes through a membrane and parasite oocysts in the mosquito midgut are enumerated approximately one week later. Using this assay, it has been shown that monoclonal and polyclonal antibodies, raised in various animal models, against P25 show transmission-blocking activity [14-16]. However, previous studies have not found a consistent relationship between antibody titer of a serum and transmission-blocking activity in the MFA. In the case of Pfs25, it is thought that there is a weak correlation [17], but other reports show there is no correlation between them [18,19]. On the other hand, a consistent relationship between transmission-blocking and anti-Pvs25 was found in a recent study [20]. Since a Phase 1 human clinical trial with Pvs25 has been conducted in the U.S. [21] and other trials are anticipated with transmission-blocking vaccines in the future, it is urgent to establish a method that can be used to evaluate MSTB vaccine potential for large numbers of samples coming from future clinical trials. As antibodies are the principal effector mechanism for vaccines against P25, it is possible that other measures of antibody titer such as ELISA might serve as a surrogate for the MFA.

MFA has been used to assess the transmission-blocking activity of antibodies directed to other mosquito stage antigens. There have been several studies that examined an association of seropositivity to Pfs48/45 or Pfs230 in human sera from endemic areas with transmission-reduction in a membrane feed (e.g. [22]). In these studies as both variables are categorical variables (either positive or negative) and as the sera may contain multiple specificities, a detailed continuous relationship between antibody and blocking cannot be derived. Partly because recombinant Pfs48/45 or Pfs230 have not been available, there have been very few studies looking at more quantitative aspects. In one study [23] that looked at the correlation between anti-Pfs48/45 and reduction in oocyst number by MFA using naturally occurring human sera, a weak but significant correlation (r^2 ^= 0.24) was found and again it is possible that the wide scatter in their data reflects other specificities in the sera. More recently, a monotonic relationship was shown between the antibody directed to single epitope (Epitope I) on Pfs48/45 in mice immunized with a refolded, recombinant fragment of Pfs48/45 [24]. However, there are still no reported studies on well characterized, total anti-Pfs48/45 activity and the quantitative effect on reduction in oocyst numbers.

Different endpoints for the MFA have been used for measuring transmission-blocking activity: a reduction in the oocyst density per mosquito or an increase in the proportion of mosquitoes that have no parasites. The former is generally used for laboratory studies, but it is the latter that is thought to best predict the likely efficacy of vaccines under field conditions. Since it is much easier to measure antibody by ELISA than to measure either a reduction in oocyst density or the decrease in malaria transmission in the field, it would be useful to understand the link between antibody titer and these two measures of transmission-blocking to facilitate vaccine development. In addition to the absolute level of the antibody, it is possible that the transmission-blocking activity may depend on the "quality" of the antibody, e.g., different fine specificities or antibody affinities resulting from maturation of the antibody response, either as a result of boosting or as a result of the use of different adjuvants. However, it is not clear whether adjuvant selection changes only the amount of antibody induced by the vaccine, or whether it also changes the quality of the antibody with respect to transmission-blocking activity.

In this paper, sources of error in measuring transmission-blocking activity were examined by evaluating the activity of sera raised in rabbits and rhesus monkey against yeast-produced Pfs25 and Pvs25, both purified to standards of clinical grade material, formulated with several adjuvants. The reduction in oocyst density and the proportion of infected mosquitoes can be directly related to the anti-P25 antibody titer in the membrane feed. For the conditions tested, different adjuvants, time after immunization or the species of animal immunized result in antibody with similar specific transmission-blocking activity.

## Methods

### Antigen preparation

Clinical grade Pfs25 was prepared as described [25]. In brief, the Pfs25 sequence from Lys-23 to Thr-193 was subcloned into the pPIC9K vector and transformed into *Pichia pastoris*. After fermentation, a series of chromatography steps was used for purification and buffer exchange as follows: Nickel nitrilotriacetic acid Superflow column, Phenyl Sepharose hydrophobic interaction chromatography and Superdex 75 size-exclusion column.

Clinical grade Pvs25 was prepared as described [26] In brief, the parasite gene encoding Pvs25 was subcloned into the plasmid YEpRPEU-3 and transformed into *Saccharomyces cerevisiae *VK1 cells. After fermentation, a series of chromatography steps was used for purification and buffer exchange as follows: Nickel nitrilotriacetic acid Superflow column, Phenyl Sepharose hydrophobic interaction chromatography and Superdex 75 size-exclusion column.

Characterization of these two products has been previously reported [25,26].

### Rabbit immunization

All rabbit studies were carried out with antisera obtained from New Zealand White rabbits (Spring Valley Laboratories, Frederick, MD). Four rabbits were immunized by intramuscular injection with 0.5 ml of saline containing 80 μg of Pfs25 adsorbed onto 800 μg of alum (Alhydrogel^®^-aluminum hydroxide; HCI Biosector, Frederikssund, Denmark) and another 4 rabbits were immunized with 80 μg of Pfs25 formulated with Montanide ISA720 (SEPPIC, Paris, France). Animals were immunized on days 0, 28 and 56, and bled on days 0 (before immunization), 28 (before boost), 42 and 70.

### Rhesus monkey immunization

This animal study was done in compliance with National Institutes of Health (NIH) guidelines and under the auspices of an Animal Care and Use Committee-approved protocol. Ten monkeys (*Macaca mulatta*, six male and four female) were randomly divided into two groups. The two groups of five animals each received: (a) 15 μg of Pvs25 adsorbed onto 600 μg of alum, or (b) 15 μg of Pvs25 adsorbed onto 600 μg of alum and 250 μg of CpG 10105 (Coley Pharmaceutical Group, Wellesley, MA). CpG 10105 has the sequence TCG TCG TTT TGT CGT TTT TTT CGA using a fully phosphorothioate backbone. The monkeys were immunized on days 0, 28 and 181 and bled on days 0 (before immunization), 42, 90 and 195.

### ELISA

Flat-bottom 96-well ELISA plates (Immunolon 4; Dynex Technology Inc., Chantilly, VA) were coated with 100 ng/well of Pfs25 or Pvs25 at 4°C overnight. The plates were blocked with 5% skim milk (Difco, Detroit, MI) in Tris Buffered Saline (TBS) (BioFluids, Camarillo, CA) for two hours at room temperature. Animal sera were diluted in buffer that contained 0.1% BSA (Sigma Chemical Co., St. Louis, MO) and 0.05% Tween 20 (Sigma Chemical Co.) in TBS. Diluted sera were added to antigen-coated wells in triplicate and incubated for two hours at room temperature. After extensive washing with 0.1% Tween 20 in TBS, the plates were incubated with the secondary antibody conjugated with alkaline phosphatase (Kirkegaard & Perry Laboratories, Inc., Gaithersburg, MD) for two hours at room temperature. Bound antibodies were visualized by adding the substrate solution (*p*-nitrophenyl phosphate Sigma 104 substrate; Sigma Chemical Co). The absorbance at 405 nm was read using a SPECTRAmax 340 PC microplate reader (Molecular Devices Co., Sunnyvale, CA).

Standardization of the ELISA was accomplished by comparison with a standard antiserum tested on each ELISA plate. Three standard sera (anti-Pfs25 serum from mice immunized with Pfs25 formulated with Montanide ISA720, anti-Pfs25 rabbit serum and anti-Pvs25 rhesus monkey serum) were prepared using sera obtained two weeks after the 2^nd ^immunization, aliquoted, and stored at -80°C until required. Serially diluted standard sera were tested and assigned unit values as the reciprocal of the dilution giving an O.D. _405 _= 1 in the ELISA. In each test ELISA plate, serially diluted standard sera were applied to prepare a standard curve. Using the standard curve, the absorbance of individual test sera was converted to antibody units (SOFTmax PRO ver.3; Molecular Devices Co., Sunnyvale, CA).

### MFA with monoclonal antibody 4B7

Anti-Pfs25 monoclonal antibody 4B7 (MAb 4B7) was assayed as described previously [18]. Briefly, the antibody was serially diluted from 1:24 to 1:384 with pooled heat inactivated human O serum, which was made from individuals who have not been previously exposed to malaria, then mixed with mature in vitro cultured *P. falciparum *gametocytes (NF54 isolate) and fed to *Anopheles stephensi *mosquitoes through a membrane-feeding apparatus. Mosquitoes were kept for 8 to 10 days after feeding to allow parasites to develop into mature oocysts. Infectivity was measured by dissecting at least 20 mosquitoes per sample, staining with Mercurochrome, and counting the number of oocysts per midgut. Within one membrane feeding experiment (i.e. a set of membrane feeds done on one day with a single shared control feed) each concentration of MAb 4B7 was tested in a single membrane feeder. The membrane feeding experiment was repeated twice on different days at four different concentrations.

The percent inhibition of oocyst density per mosquito was determined by the formula: 100 - (arithmetic mean of oocysts in the test sample/arithmetic mean of oocysts in pooled human O serum) × 100.

The anti-Pfs25 antibody units used in the membrane feeding apparatus for each dilution of MAb 4B7 were calculated based on the ELISA-determined antibody units of the original MAb and the dilution factor of the feeding sample.

### MFA with rabbit sera

Anti-Pfs25 rabbit sera were tested with cultured *P. falciparum *gametocytes in a similar fashion to the MAb 4B7. Six pooled sera were made from: 1) Day 0 sera of 4 rabbits immunized with Pfs25 and alum (alum group), 2) Day 0 sera of 4 rabbits immunized with Pfs25 and ISA720 (ISA group), 3) Day 42 sera of alum group, 4) Day 42 sera of ISA group, 5) Day 70 sera of alum group, 6) Day 70 sera of ISA group. The pooled sera and individual rabbit sera were heat inactivated, diluted with the pooled heat inactivated human O serum at 1:2 and 1:8 dilutions, then mixed with the *P. falciparum *gametocytes and fed to mosquitoes as above. In each membrane feeding experiment, the pooled or individual sera were tested in a single membrane feeding apparatus at 1:2 and 1:8 dilution. A limited set of the sera were retested on three occasions.

Percent inhibition of oocyst density per mosquito was determined by the formula: 100 - (arithmetic mean of oocysts in the test sample/arithmetic mean of oocysts in pooled day 0 serum for that particular adjuvant) × 100.

Percent inhibition of infected mosquitoes was determined by the formula: 100 - (percent of infected mosquitoes in the test sample/percent infected mosquitoes in pooled day 0 serum for that particular adjuvant) × 100.

As for the MAb 4B7, the anti-Pfs25 antibody units in each membrane feeding test sample were calculated based on the ELISA-determined antibody units in the original serum and the dilution factor of the feeding sample.

### MFA with *P. vivax *in Thailand

Anti-Pvs25 rhesus monkey sera were tested for transmission-blocking activity with *P. vivax *infected human blood as a source of gametocytes. Informed consent was obtained from all volunteers. Volunteers came to malaria clinics in north-west Thailand and were diagnosed with *P. vivax *infection microscopically. Blood was collected in heparin. Less than two hours after collection, blood was centrifuged and the plasma was removed. Mixtures of packed erythrocytes and heat inactivated test sera, which were diluted with a pool of heat inactivated American normal human AB+ serum (prepared from malaria-naïve donors) as 1:2 or 1:8 dilutions, were made. Mixtures were immediately placed in feeding apparatuses and offered to *Anopheles dirus*. These mosquitoes were individually separated to remove unfed mosquitoes and incubated at 28°C for seven to nine days before dissection of mosquito midguts. The oocysts formed in the midgut were examined and counted by microscopy. The pooled normal heat inactivated human AB serum was used as an indicator of a successful feed for each set of rhesus sera and for each parasite source. If the arithmetic mean of oocyst numbers for the human AB sera was less than four in 20 to 40 dissected mosquitoes, the feeding result was discarded. Each sample was tested with parasites from different patients, until at least three successful feeds were achieved. Only data sets which showed an arithmetic mean number of oocysts for the day 0 serum (both 1:2 and 1:8 dilutions) over four, were used to calculate the transmission-blocking activity for each test serum.

Percent inhibition of oocyst density per mosquito was determined by the formula: 100 - (arithmetic mean of oocysts in the test for a particular monkey/arithmetic mean of oocysts in the day 0 serum for that same monkey and for that particular dilution) × 100.

Anti-Pvs25 antibody units of a feeding sample were calculated based on the ELISA-determined antibody units in the original serum and the dilution factor of the feeding sample.

### Statistics

For comparing antibody units of two groups, the Mann-Whitney U-test was performed. To test the correlation between units of antibody and transmission-blocking activity, a Spearman rank correlation was used. Statistical analyses were done by UNISTAT 5.0 (P-STAT Inc., Hopewell, NJ). Probability values less than 0.05 are considered as significant. Curve fitting analyses were performed using Sigma Plot (SPSS Inc., Chicago, IL) and the regression analysis used to calculate the concentration of antibody giving 50% inhibition of oocyst numbers (Ab_50_) and its 95% confidence limit. Note that this Ab_50 _value is calculated from the whole data and its use is analogous to the use of IC50 values for drug studies. The antibody required to give 80% inhibition was also calculated from the fitted regression analysis as a value indicating a level of antibody that could be expected to have a biological impact.

### Computer simulations model of the MFA

Two computer simulations models were written to evaluate sources of experimental errors in correlating antibody levels with the percent inhibition of oocyst density per mosquito and to examine the relationship between reduction in oocyst numbers and the proportion of mosquitoes with no infection. Data sets were assembled from experimental data on the oocyst density in individual mosquitoes measured in control feeds (i.e., mosquitoes feeding on gametocytes but with no added antibody). Two *P. falciparum *data sets were obtained from the two experiments to measure MFA activity in rabbit anti-Pfs25 sera described above. Data sets were also obtained from control feeds using *P. vivax *blood from volunteers. These data were obtained during the experiments to measure transmission-blocking activity with rhesus anti-Pvs25 sera. Details of the data sets are listed in Table 1. Both sets of *P. falciparum *data had similar mean oocyst numbers. However, the computer simulation picks values for the entire range (0 to 131) oocysts per mosquito, so a much larger range is sampled than indicated from these mean values. Similarly, the *P. vivax *data cover a range of 0 to 605 oocysts per mosquito.

**Table 1:** Experimental data sets used for simulation studies

Data Set	No. Mos.^a^	Mean Ooc.^b^	C.V.^c^	% Uninf.^d^
***Rabbit anti-Pfs25***				
Pf1	81	28.05	1.00	7
Pf2	94	28.99	0.63	0
				
***Rhesus anti-Pvs25***				
Pv1	140	6.66	1.10	34
Pv2	160	8.56	0.95	23
Pv3	146	13.50	1.15	33
Pv4	168	17.14	0.90	14
Pv5	140	22.56	0.90	21
Pv6	160	34.21	0.92	19
Pv7	155	56.18	0.99	22
Pv8	175	93.89	0.96	25
Pv9	178	135.35	0.96	28
Pv10	119	231.10	0.65	11

^a ^Number of dissected mosquitoes.

^b ^Mean number of oocysts per mosquito.

^c ^Coefficient of variation in mean.

^d ^Percent of uninfected mosquitoes.

The first programme was used to test the relationship between reduction in average oocyst numbers and reduction in the proportion of mosquitoes infected. Specifically it tested if the proportion of infected mosquitoes in a test feed can be predicted from the measured survival in individual feeds and the observed distribution of oocyst densities in control feeds. It assumes that the survival of each oocyst is independent of the distribution of oocysts in the mosquito population, i.e., it assumes that an antibody that gives a 50% reduction in oocyst density in a mosquito population with an average oocyst density of for example 6.6 per mosquito will also give a 50% reduction if the control mosquitoes have an average of 230 per mosquito. For each datum point in the simulation, the programme used the number of mosquitoes dissected in an actual control feed (*nc*), the number of test mosquitoes dissected (*nt*) and the observed oocyst density in each test feed. The ratio of experimentally determined oocyst densities in test to control feeds was used as a measure of the probability of oocyst survival (*ps*) in the presence of antibody. In experimental feeds where the number of oocysts in the test feeds was greater than in control, *ps *was set to 1.0. The programme randomly selected *nc *mosquitoes as the simulated control and *nt *mosquitoes as the simulated test group from the appropriate data set. For each of the mosquitoes in the test group, the simulated number of oocysts was calculated using a binomially distributed random number, with probability *ps*, then the % infected mosquitoes in the test group was calculated. The simulation was run for each of the experimental feeds using *P. falciparum *blood in the presence of rabbit anti-Pfs25 and for each feed using *P. vivax *infected blood in the presence of Rhesus anti-Pvs25. The simulation was repeated 40 times for each experimental data point.

The second programme was used to simulate the relationship between antibody concentration and % inhibition of oocyst numbers. The programme was identical, except that the probability of survival, *ps*, used for each simulation was calculated from the experimentally determined hyperbolic best fit correlating % inhibition of oocyst density with antibody concentration.

## Results

### Anti-Pfs25 monoclonal antibody 4B7 has potent transmission-blocking activity

**Figure 1:**
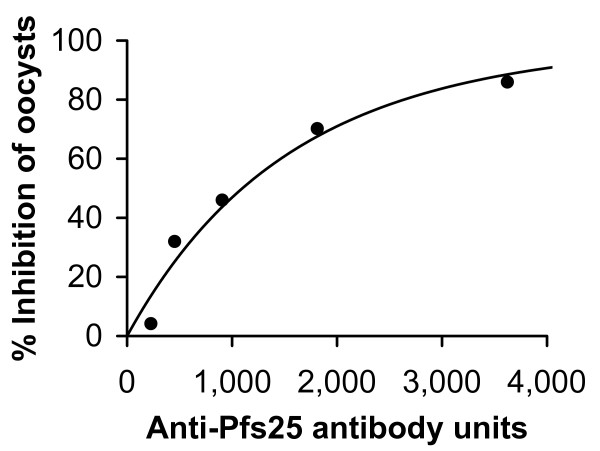
**Concentration-dependent TB activity of anti-Pfs25 monoclonal antibody 4B7**. MAb 4B7 was serially diluted with pooled human O serum to give the antibody concentration in the feed as indicated (X-axis), mixed with mature in vitro-cultured *P. falciparum *gametocytes and fed to *A. stephensi *mosquitoes. Approximately one week later, oocysts were counted and % inhibition of oocyst density per mosquito relative to mosquitoes fed with a pooled human O serum is plotted (Y-axis). Line represents regression of result by use of a hyperbolic equation.

To study the relationship between antibody concentration and transmission-blocking activity for *P. falciparum*, the relationship between the transmission-blocking activity of a monoclonal antibody and its concentration determined by ELISA was investigated. Anti-Pfs25 monoclonal antibody 4B7 (MAb 4B7) has been previously described by Kaslow and colleagues as being directed to Pfs25 and having transmission-blocking activity in MFA [14]. The MAb 4B7 was serially diluted and the samples were used in MFA. As shown in Figure 1 for one of the three feeding experiments, MAb 4B7 showed concentration-dependent transmission-blocking activity; a plot of % inhibition of oocyst density per mosquito versus antibody concentration determined by ELISA followed a hyperbolic curve (r^2 ^= 0.945): % inhibition = 100-100/(1+4.69 × 10^-5 ^× ELISA units)^13.8^. This curve gives a 50% inhibition at 1038 units (95% CI range 677 to 1562). The other two feeding experiments gave similar good fits (r^2 ^= 0.967 and 0.903). They used a narrower range of antibody concentrations (four 2-fold dilutions each instead of five) and gave Ab_50 _of 477 (312 to 720) and 897 (335 to 2169), respectively. Note that the confidence intervals reflect intra-assay variation only. Errors in the determination of the number of oocysts in the control group will cause a systematic error in all of the calculated % errors within one experiment and will have a small impact on the fit within one experiment but they will contribute to the inter-assay variation.

### Pfs25 induced transmission-blocking antibody in rabbits

**Figure 2:**
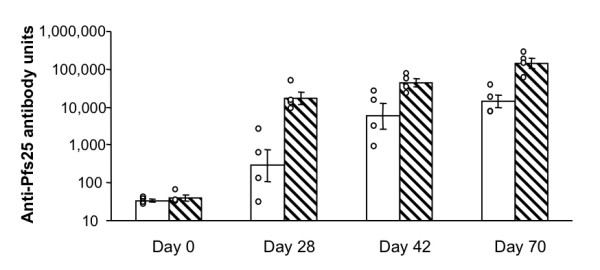
**Kinetics of induction of rabbit anti-Pfs25 antibody**. Groups of 4 rabbits were immunized with (i) 80 μg of Pfs25 adsorbed onto alum (blank bars), or (ii) 80 μg of Pfs25 formulated with Montanide ISA720 (hatched bars). Animals were immunized on days 0, 28 and 56, and bled on days 0, 28, 42 and 70. Sera were tested for anti-Pfs25 antibody concentration by ELISA relative to a standard rabbit anti-Pfs25 serum which has 100,000 units. ELISA values of individual rabbits are plotted and the geometric mean ± SE for the group are also shown. On day 70, the antibody units of the Pfs25-ISA group are significantly higher than those of the Pfs25-alum group (Mann-Whitney U-test, p < 0.0001).

To extend these results to polyclonal antisera and to another species, groups of four rabbits were immunized at day 0 and 28 with either Pfs25 adsorbed onto alum (Pfs25-alum group) or Pfs25 formulated with Montanide ISA720 (Pfs25-ISA group). Day 0 sera from both groups showed negligible anti-Pfs25 antibody measured by ELISA (Figure 2). Two weeks (day 42) after the second immunization, all animals made significant levels of anti-Pfs25 antibodies. The two different groups showed geometric means (GM) of 5,831 units for the Pfs25-alum group and 43,983 units for the Pfs25-ISA group. Differences between the two groups in ELISA titer do not reach statistical significance (Mann-Whitney U-test, p = 0.057). On day 70, the GM of the Pfs25-ISA group was 144,973 and that of Pfs25-alum group was 14,202, and at this time point the antibody units of the Pfs25-ISA group are significantly higher than those of the Pfs25-alum group (Mann-Whitney U-test, p < 0.0001).

**Figure 3:**
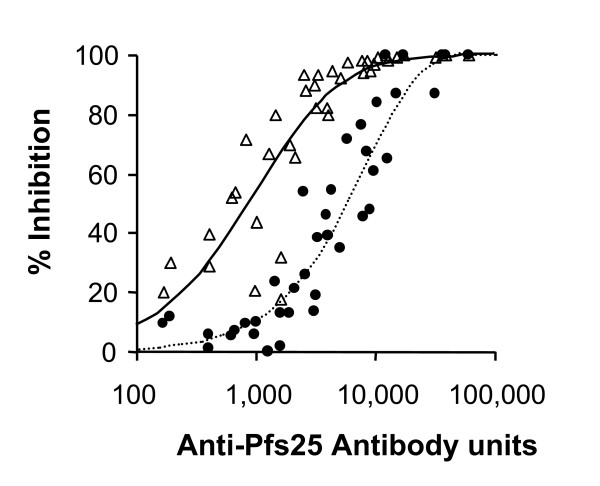
**Correlation of TB activity and antibody units of rabbit anti-Pfs25 sera**. Groups of 4 rabbits were immunized with 80 μg of Pfs25 as described in the legend to Figure 2. Individual and pooled sera were diluted 1:2 and 1:8, and then tested for TB activity in mosquito membrane feeding assays using sexual stages of *P. falciparum *and *A. stephensi *mosquitoes. Anti-Pfs25 antibody units of each sample prior to dilution were determined by ELISA. Antibody unit values for the diluted sera are plotted on the X-axis and % inhibition of oocyst density per mosquito (open triangles) of the same sample or % inhibition of infected mosquitoes (closed circles) of the same sample is plotted on the Y-axis. There is a strong correlation between the antibody units and % inhibition of oocyst density per mosquito or % inhibition of infected mosquitoes (the Spearman Rank Correlations are 0.934 and 0.900, respectively). Lines represent regression of result by use of a hyperbolic equation: Solid line – correlation between antibody units and % inhibition of oocyst density per mosquito; broken line – correlation between antibody units and % inhibition of infected mosquitoes.

The biological activities of individual and pooled rabbit sera were then tested with mature in vitro-cultured *P. falciparum *gametocytes by MFA. As shown in Figure 3, there is a strong correlation between the antibody ELISA units and % inhibition of oocyst density per mosquito; the Spearman Rank Correlation (*r*_*s*_) is 0.934 (95% CI: 0.878–0.965, p < 0.0001). The relationship followed a hyperbolic curve (r^2 ^= 0.697): % inhibition = 100-100/(1+6.24 × 10^-4 ^× ELISA units)^1.59 ^giving 50% inhibition at 877 units (CI 629 to 1212) based on two membrane feeding experiments each with one control feed and 20 test feeds (n = 40) Based on this hyperbolic curve, 80% inhibitions of oocyst density per mosquito were achieved with approximately 2,800 antibody ELISA units. A limited set of the sera were tested on three other occasions giving Ab_50 _of 594 (CI 428 to 816, r^2 ^= 0.782, n = 11, one membrane feeding experiment); 2100 (CI 1009 to 3969, r^2 ^= 0.663, n = 14, two membrane feeding experiments with eight and six test feeds, respectively).

Transmission-blocking activity in MFA can also be evaluated by determining the proportion of infected mosquitoes. The relationship between the antibody ELISA units and % inhibition of infected mosquitoes was also strong (*r*_*s *_= 0.900, 95% CI: 0.818–0.946, p < 0.0001), and the relationship followed a hyperbolic curve (r^2 ^= 0.858): % inhibition = 100-100/(1+1.29 × 10^-6 ^× ELISA units)^97.5^. However, the % inhibition of infected mosquitoes was less than the % inhibition of oocyst density per mosquito when tested at the same antibody concentration.

**Figure 4:**
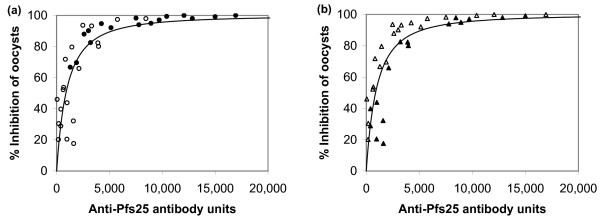
**Effect of adjuvant selection and time after immunization on TB activity**. Individual and pooled rabbit anti-Pfs25 sera were diluted 1:2 and 1:8, and then tested for TB activity in mosquito membrane feeding assays as described in the legend to Figure 3. (a) data were categorized according to the adjuvant used for immunization (open circles: alum; closed circles: Montanide ISA720) while in (b) data were categorized according to number of days after initial immunization (open triangles: day 42; closed triangles: day 70). Lines represent regression of result by use of a hyperbolic equation. Four data points, which showed more than 30,000 antibody units and 100% inhibition of oocyst density per mosquito, are not shown in these figures.

As part of this study, the impact of the selection of adjuvant or the number of immunizations on the inhibitory activity of the sera was also examined. Regardless of the adjuvant utilized (Figure 4a), or the number of days after immunization (Figure 4b), all data points followed the same hyperbolic curve when the % inhibition of oocyst density per mosquito was plotted against antibody units.

### Pvs25 induced transmission-blocking antibodies in rhesus monkeys

**Figure 5:**
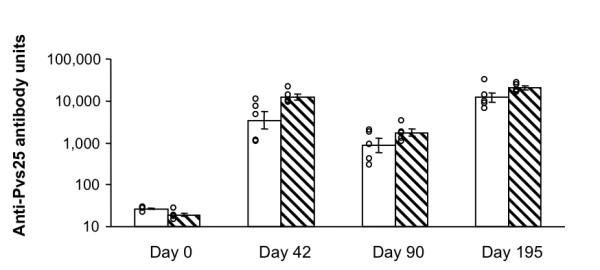
**Kinetics of antibody response to immunization with Pvs25 in rhesus monkeys**. Groups of 5 monkeys were immunized with (i) 15 μg of Pvs25 adsorbed onto 600 μg of alum (blank bars), or (ii) 15 μg of Pvs25 adsorbed onto 600 μg of alum and 250 μg of CpG 10105 (hatched bars). Animals were immunized on days 0, 28 and 181, and bled on days 0, 42, 90 and 195. All sera were tested by ELISA and compared to a standard rhesus anti-Pvs25 serum which has 20,000 units. ELISA values of individual monkeys are plotted and the geometric mean ± SE for the group are also shown.

These results were extended to *P. vivax*. Five rhesus monkeys (*Macaca mulatta*) were immunized with the *vivax *homologue of Pfs25, i.e., clinical grade Pvs25 adsorbed onto alum (Pvs25-alum group). A second group of 5 monkeys was immunized with Pvs25 adsorbed onto alum with the addition of CpG10105 (Pvs25-CpG group). As shown in Figure 5, there was a negligible concentration of specific antibodies in the monkey sera before immunization (day 0), but by two weeks (day 42) after the second immunization, Pvs25 elicited significantly higher antibody titers for both the Pvs25-alum group (GM was 3,436 units) and the Pvs25-CpG group (12,351 units). By day 90, the antibody units had declined in both groups (GM of the Pvs25-alum group was 589 and the Pvs25-CpG group was 1,738). Two weeks (day 195) after a third immunization, the antibody values exceeded the day 42 antisera (GM of the Pvs25-alum group was 12,044 and the Pvs25-CpG group was 20,392). The GM of the Pvs25-CpG group was higher than that of the Pvs25-alum group on all days after immunization, but there were no statistically significant differences using a Mann-Whitney U-test.

**Figure 6:**
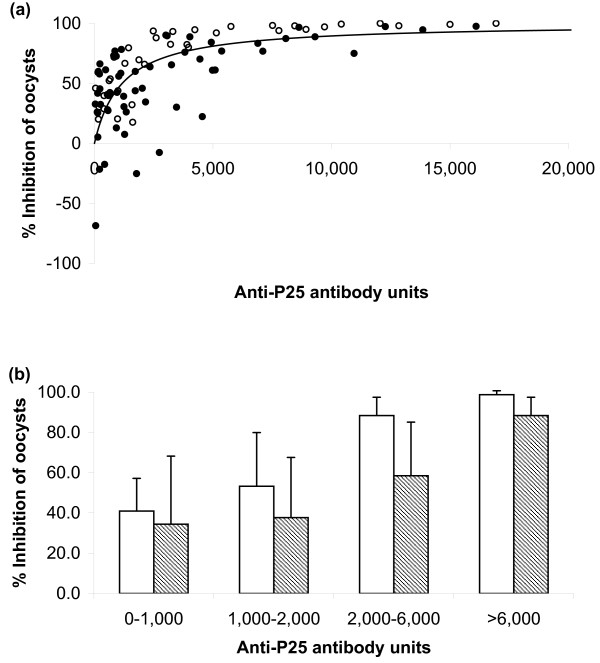
**Correlation of TB activity with antibody titer using anti-Pfs25 sera from rabbits and anti-Pvs25 sera from rhesus monkeys**. Rabbits were immunized with Pfs25 and the antisera were tested in mosquito membrane feeding assays with *P. falciparum *parasites and *A. stephensi *mosquitoes as described in legend to Figure 3. Rhesus monkeys were immunized with Pvs25 as described in legend to Figure 5 and the antiserum was diluted with a pool of normal human AB+ serum as 1:2 or 1:8 dilutions. The diluted monkey sera were tested in membrane feeding assays using parasites derived from *vivax*-infected patients in Thailand and *A. dirus *mosquitoes. Antibody units in the sera were determined by standardized ELISA. (a): Rabbit data (open circles) and individual monkey data (closed circles) are shown. There is a significant correlation between antibody units and % inhibition of oocyst density per mosquito in rabbit and in monkey sera (the Spearman Rank Correlations are 0.934 and 0.616, respectively). Lines represent regression of monkey result by use of a hyperbolic equation. Four points of rabbit data, which showed more than 30,000 antibody units and 100% inhibition of oocysts, are not shown in this figure. (b):The values of the test sera were grouped as shown. Average percent oocyst inhibition for rabbit sera (blank bars) or monkey sera (hatched bars) in each ELISA range + SD are shown.

Figure 6 shows the MFA results with these monkey sera and the relationship with antibody titer as determined by ELISA. As in the case of the data obtained with rabbit anti-Pfs25 sera, it is apparent that there is a significant correlation between antibody ELISA units and % inhibition of oocyst density per mosquito in rhesus monkey sera (*r*_*s *_= 0.616, 95%CI: 0.429–0.752, p < 0.0001). Moreover, the data points followed a hyperbolic curve (r^2 ^= 0.249): % inhibition = 100 × ELISA units/(1567 + ELISA units) when the % inhibition of oocyst density per mosquito was plotted against antibody units. Based on the fitted hyperbolic curve, 50 and 80% inhibitions were achieved with approximately 1,500 and 6,000 antibody units, respectively.

### Computer simulation of MFA

**Figure 7:**
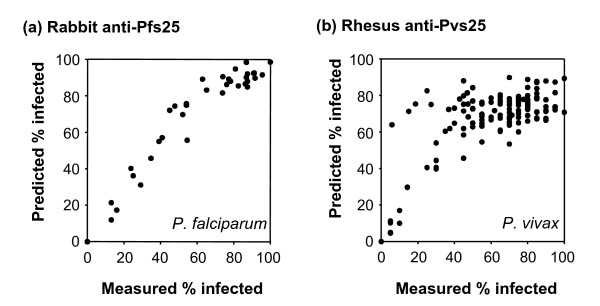
**Comparison of predicted and measured percentage of infected mosquitoes**. Predicted values are the average of 40 computer simulations per point for membrane feeds based on the experimental feeds measuring TB activity of rabbit anti-Pfs25 sera (a) and rhesus anti-Pvs25 sera (b). These are the same feeds shown in Figure 4 and Figure 6, respectively.

For both *P. falciparum *and *P. vivax*, the computer simulations based on the reduction in oocyst density gave a good fit between the predicted and experimentally measured proportion of mosquitoes infected (Figure 7). For *P. falciparum*, the Spearman rank correlation, *r*_*s *_was 0.937 for the correlation of mean % infected mosquitoes from simulated data with the experimental data and 0.967 for the correlation of mean % infected from simulated and a single simulated data set. This suggests that the model not only gave the expected relationship but also closely predicted the variance in the experimental data. For *P. vivax *the fit was not as good with *r*_*s *_values of 0.465 and 0.675, respectively, and the simulated average % infected mosquitoes vs. experimental % infected deviated from the expected linear relationship at high % infected.

**Figure 8:**
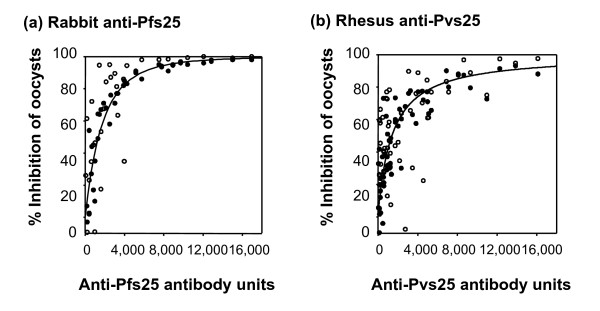
**Comparison of predicted and measured oocyst density per mosquitoes**. Experimental data (open circles) are shown for anti-Pfs25 (a) and anti-Pvs25 (b) and are the same feeds shown in Figure 4 and Figure 6, respectively. A single simulated data set (closed circles) is shown for each.

In the simulation of oocyst density as a function of antibody concentration, the computer simulations showed that the expected errors from sampling were substantially lower than the observed fit (Figure 8). The r^2 ^for *P. falciparum *hyperbolic fit was 0.697 for experimental data and the mean r^2 ^for the simulated data was 0.899. For *P. vivax*, the respective values were 0.249 and 0.633.

## Discussion

This is the first study that clearly shows that the biological activity of transmission-blocking antisera is a direct function of antibody titer. There are two reasons that can be suggested to account for the correlation observed in this study. One reason may be the way that the MFA were performed, particularly with respect to the number of mosquitoes analysed and the number of samples that were used. The second reason may be the precision with which the antibody titers were determined in a standardized ELISA format.

For transmission-blocking immunity measured as a decrease in the oocyst density per mosquito, a reproducible hyperbolic relationship between antibody concentration measured by ELISA and the level of inhibition was observed. Part of this evaluation tested if antibody concentration alone was the major determinant of transmission-blocking activity or whether other characteristics of antibodies might also affect the biological activity of the sera. To address this question, the selection of adjuvant and the time the sera was collected after immunization was assessed to see the impact on ELISA units and transmission-blocking activity tested by MFA. Aluminum salts (alum) are commonly used in vaccines in clinical use although it is a relatively weak adjuvant for antibody induction in humans [27,28]; such formulations have been reported to give primarily a Th2 response in mice [29]. Montanide ISA720, a water-in-oil emulsion adjuvant, has been used with malarial vaccine candidates both in animal studies [30-34] and in human trials [35-37]. Unmethylated CpG dinucleotides, which are found frequently in genomes of bacteria and viruses, trigger an innate immune response [38] and also stimulate B cell proliferation and immunoglobulin secretion [39]. Oligonucleotides containing CpG motifs have been shown to be strong adjuvants for DNA- or protein-based immunogens [40-43] and have been used for human trials [44,45]; they elicit primarily a Th1 response in mice [40,46].

In the case of rabbits immunized with Pfs25, adjuvant selection and the additional times of immunization significantly changed the quantity of antibody produced (Figure 2), but did not change the quality of the antibody with respect to transmission-blocking activity when normalized for antibody concentration (Figure 4). The data clearly shows that regardless of adjuvant or time after immunization, all transmission-blocking data followed the same hyperbolic curve, indicating that antibody titer was the major determinant of biological activity. In the case of rhesus monkeys immunized with Pvs25, no statistically significant differences in ELISA values between the Pvs25-alum group and the Pvs25-CpG group could be detected at any time point (Figure 5). Although the GM of antibody units of the Pvs25-CpG group were always higher than those of the Pvs25-alum group on all days, including days 14, 28, 175, 223, 269 and 365 (data not shown), the small group sizes preclude a definitive test of significance using the non-parametric Mann-Whitney U-test. Like the rabbit data, the MFA data of monkeys showed that neither adjuvant selection nor time of sera collection changed the transmission-blocking activity when it was normalized for antibody concentration (data not shown). Based on these results, a vaccine formulation that shows the highest immunogenicity judged by ELISA should be selected for a human anti-P25 vaccine, assuming that there are no safety concerns.

Surprisingly, regardless of the parasite antigen (Pfs25 or Pvs25) or the species producing the antibodies (rabbit or monkey) or the species of mosquito (*A. stephensi *or *A. dirus*) used in the MFA, similar transmission-blocking activity was obtained when the % inhibition of oocyst density per mosquito was plotted against antibody ELISA units (Figure 6a). In addition, the anti-Pfs25 monoclonal antibody 4B7, produced by a murine hybridoma, also displayed the same hyperbolic curve as the rabbit anti-Pfs25 sera (Figures 1 and 6). Approximately 1,000 (for anti-Pfs25) or 1,500 (for anti-Pvs25) units of antibody gives 50% inhibition of oocyst density per mosquito and 80% inhibition can be achieved with approximately 3,000 (for anti-Pfs25) or 6,000 (for anti-Pvs25) antibody units. Since the data with *P. vivax *were scattered, it is not possible to judge whether anti-Pfs25 sera had really higher transmission-blocking potency than that of anti-Pvs25 sera. Overall, these data were surprisingly close considering the many inherent variables in this biological assay and the many differences intrinsic to the two assay systems. The important point of these studies is the support for the hypothesis that the same quantity of antibody will be required to achieve a desired level of transmission-blocking activity in human clinical trials with P25.

The reduction in the number of mosquitoes also had a simple relationship with antibody. However, as mosquitoes usually had many oocysts, the concentration of antibody required to give a 50% reduction in the number of infected mosquitoes was much higher than the amount required to kill 50% of the oocysts. Again as expected, from the need to kill multiple oocysts, the shape of the relationship was also different: where as the curve for percent killing was close to a simple rectangular hyperbola (exponent of 1.57 in the two parameter fit compared to 1.0 for a simple rectangular hyperbola), the relationship between antibody and % uninfected mosquitoes was much steeper (exponent 97.5) consistent with the need to independently kill multiple oocysts.

Because the MFA is a complex, time and labor-consuming assay, most of the MSTB vaccine studies have used 30 or fewer mosquitoes per sample [14-16,18,19,47]. However, transmission-blocking activity determined from small numbers of mosquitoes per feed are likely to lead to large sampling errors, because the distribution of oocysts per mosquito is highly over dispersed (Table 1). With the MFA reported in this paper for *P. falciparum*, the average oocyst density per mosquito was similar for each of the control feeds. However, this is unusual. In general, wide variations could be expected from one experiment to another. Actually, in infections resulting from mosquitoes directly feeding on infected humans, large variations in oocyst numbers have also been reported [48]. This was particularly the case for the *P. vivax *feeds. Approximately half of the feeds had less than 4 oocysts per mosquito (these were not analysed), and the remainder varied from an average of 6.6 to 230. Since *P. vivax *cannot be cultured continuously and the only source of the gametocytes is infected non-human primates or humans, it is difficult to control the quality of parasites for the assay. Therefore, the MFA data obtained with *P. vivax *were more scattered than the data tested with culture-derived *P. falciparum *gametocytes (Figure 6). Presumably in these cases, in which zero or few oocysts per mosquito were observed, the gametocytes were in insufficient quantity or inappropriate state of maturation to give a productive mosquito infection.

Because of the dispersion, Medley et al suggested that less than 100 mosquitoes per feed, especially less than 50 per feed, provide unreliable estimates of transmission-blocking activity [49]. An estimate of the contribution of this over dispersed distribution can be obtained from the computer simulations of MFA. For the simulation models, the oocyst distributions in control mosquitoes and the numbers of mosquitoes dissected in control and test groups were used to obtain the experimental data. Thus the scatter in the simulated data only includes variation due to sampling. However, these simulations based on actual control feeds, showed that even with 20 mosquitoes per feed, the variation due to the sampling error is small compared to other as yet other unidentified variations between replicate feeds (Figure 8). This is similar to the results recently reported from another study of oocyst distributions obtained from membrane feed data using cultured *P. falciparum *gametocytes [50]. These results show that reliable estimates of transmission-blocking activity require multiple feeds per sample with relatively small numbers of mosquitoes rather than a single feed with large numbers of mosquitoes. In this paper, each rhesus serum was tested with *P. vivax *from at least three different patients. When the average % inhibition was calculated as described in Materials and Methods, there was a significant correlation between antibody titer and the transmission-blocking activity.

Depending on the underlying biological relationship between antibody level and killing of parasites, one may expect that for a given antibody level, the proportion of mosquitoes that are prevented from becoming infected may be very different if control mosquitoes are infected by an average of say two oocysts or 230 oocysts. The computer simulation supports this conclusion. The model used assumes that the proportion of uninfected mosquitoes can be predicted from the distribution of oocysts in control mosquitoes, the probability that each oocyst will be killed is fixed by the feeding conditions and is independent of the oocyst distribution. The fit between the simulated and actual data sets for *P. vivax *is important since the input data on which this simulation is based contained control feeds with a wide range of oocysts per mosquito. Although the overall fit was good, for *P. vivax *this simple model deviated from ideal in two ways. There were a number of outliers at low proportions of mosquitoes infected (Figure 7b). Examination of these individual data points showed that they were derived from experimental feeds where the estimated probability of survival had large errors. For example, in Figure 7b, the data point (5.9, 64) is from an experimental feed that resulted in a 1/19 mosquitoes being infected and this infected mosquito had 40 oocysts. The model also deviated from observed for feeds where the test feeds had negligible transmission-blocking activity (i.e., for experimental data with a high % infected). At least part of this is due to an artifact introduced to simplify the model: experimentally derived values of inhibition were used as the basis of the model. At low antibody levels, random fluctuations in the number of oocysts result in some samples with an apparent enhancement of oocyst numbers or % of mosquitoes infected. As the model did not allow an apparent enhancement, (probability of survival must be <= 1.0), this leads to a systematic error for those pairs of experimental and simulated data at high infectivities and particularly where the actual number of infected mosquitoes is greater than the average number in the control groups.

## Conclusion

Notwithstanding the limits of such a simple model, the results presented in this paper show that within the limits of the MFA, the reduction in the oocyst density per mosquito can be predicted from the concentration of anti-P25 antibody and that this reduction is largely independent of the species from which the antibody is derived, or from the formulation and adjuvants used to generate the antibody. Furthermore, the proportion of mosquitoes that fail to become infected can also be predicted from the distribution of oocysts in control mosquitoes and from the % reduction in the oocyst density in the presence of antibody. Since the rationale for developing MSTB vaccines is to reduce the number of infected mosquitoes in the field, there is now a theoretical basis for linking antibody production in Phase 1 and Phase 2 vaccine trials to predicted reduction in transmission. While this relationship will have to be confirmed at each stage through the development of a vaccine, the prospect that ELISA values can at least in part replace cumbersome MFA as measure of transmission-blocking activity will greatly facilitate future trials of this and other mosquito stage vaccine candidates. Moreover, it also establishes a benchmark for the antibody levels which must be achieved in order to attain a defined level of transmission-blocking activity. These studies thus provide strong support that transmission-blocking activity for both *P. falciparum *and *P. vivax *parasite antigens P25 is a direct and predictable function of antibody concentration.

## Authors' contributions

KM planned and undertook the vaccinations performed the immunoassays, analysed data and drafted the paper. DK and OM carried out the membrane feeding assays with *P. falciparum*. JS carried out the membrane feeding assays with *P. vivax*. The work was undertaken in CL's laboratory and she was involved with the data analysis and paper drafting. AS developed the computer models and statistical analyses and drafted the paper.
